# Antioxidant and antiproliferative activity of blue corn and tortilla from native maize

**DOI:** 10.1186/s13065-017-0341-x

**Published:** 2017-10-30

**Authors:** Mónica Y. Herrera-Sotero, Carlos D. Cruz-Hernández, Carolina Trujillo-Carretero, Mauricio Rodríguez-Dorantes, Hugo S. García-Galindo, José L. Chávez-Servia, Rosa M. Oliart-Ros, Rosa I. Guzmán-Gerónimo

**Affiliations:** 1 UNIDA, Instituto Tecnológico de Veracruz, M.A. de Quevedo 2779, Col. Formando Hogar, 91897 Veracruz, Veracruz Mexico; 2 INMEGEN, Periférico Sur No. 4809, Col. Arenal Tepepan, Delegación Tlalpan, C.P. 14610 Ciudad de Mexico, Mexico; 3 Instituto de Ciencias Básicas, Universidad Veracruzana, Av. Dr. Luis Castelazo Ayala s/n Col. Industrial Ánimas, 91190 Xalapa, Veracruz Mexico; 4 Centro Interdisciplinario de Investigación para el Desarrollo Integral Regional del Instituto Politécnico Nacional-Unidad Oaxaca, Calle Hornos No. 1003, 71230 Santa Cruz Xoxocotlán, Oaxaca Mexico

**Keywords:** Blue corn, Tortilla, Antioxidant, Antiproliferative, Anthocyanins

## Abstract

**Background:**

Blue corn is a cereal rich in phenolic compounds used to make blue tortillas. Tortillas are an important part of the Mexican diet. Blue corn and tortilla represent an important source of the natural antioxidants anthocyanins. However, studies on their biological activity on cancer cell lines are limited. The goal of this study was to evaluate the antioxidant and antiproliferative activity of blue corn and tortilla on different cancer cell lines.

**Methods:**

Total polyphenol content, monomeric anthocyanins, and antioxidant activity by the DPPH and TBARS methods of blue corn and tortilla were determined. The anthocyanin profile of tortilla was obtained by means of HPLC–ESI-MS. The antiproliferative activity of blue corn and tortilla extract on HepG2, H-460, Hela, MCF-7 and PC-3 was evaluated by the MTT assay.

**Results:**

Blue corn had higher content of total polyphenols and monomeric anthocyanins as well as lower percentage of polymeric color than tortilla; however, both showed similar antioxidant activity by DPPH. In addition, although a higher degradation of anthocyanins was observed on tortilla extract, both extracts inhibited lipid peroxidation (IC50) at a similar concentration. The anthocyanin profile showed 28 compounds which are primarily derived from cyanidin, including acylated anthocyanins and proanthocyanidins. Blue corn and tortilla extracts showed antiproliferative effects against HepG2, H-460, MCF-7 and PC-3 cells at 1000 μg/mL, however Hela cells were more sensitive at this concentration.

**Conclusion:**

This is the first report to demonstrate anticancer properties in vitro of tortilla derived from blue corn, suggesting that this product has beneficial health effects. In addition, blue corn could be a potential source of nutraceuticals with anticancer activity.

## Background

Epidemiological studies from several countries point out that consumption of fruits, vegetables and cereals reduce the risk of chronic degenerative diseases due to the presence of bioactive compounds such as phenolic compounds [1, 2]. In recent years, pigmented cereals such as red, purple and black rice, black sorghum, and blue or purple maize have been the focus of scientific studies since they are a potential source of anthocyanins [3].

Mexico is the center of origin and biodiversity of maize (*Zea mays* L.). Species have an extensive genetic diversity, with 59 different races described with different shapes and colors ranging from white to yellow, red, purple and blue [4]. Maize (*Zea mays* L*.)* is the most important cereal in Mexico from which tortilla is produced. Tortillas are a staple food for Mexicans, consumed by 94% of the Mexican population, with a 335 g/day consumption per capita, equivalent to 122 kg/year [5].

In recent years, tortillas produced from blue maize have been the focus of scientific studies due to their anthocyanin content [6, 7]. From the chemical standpoint, anthocyanins are phenolic substances that belong to the group of flavonoids derived from the 2-phenylbenzopyrilic cation that is found in nature in a glycosylated or acylated form [2]. Several studies indicate that anthocyanins have antioxidant and anticancer properties [8, 9]. However, their biological properties are affected by food processing conditions. Nixtamalization, also known as alkaline cooking, is the traditional process for making corn dough used to prepare tortillas. From the nutritional point of view, nixtamalization has several benefits: increases calcium content, makes niacin available and reduces the amount of mycotoxins present in maize [10].

On the other hand, nixtamalization causes degradation of anthocyanins. Several studies on the effect of nixtamalization on blue tortilla have been focused on the changes of total content, profile, and antioxidant activity of anthocyanins [11, 12]. However, the number of studies related to the anticancer activity of blue corn and tortilla is limited. Given the above, the aim of this study was to determine the antioxidant properties and antiproliferative activities of blue corn and tortilla from native maize on liver, lung, cervix, breast and prostate cancer cell lines.

## Experimental

### Plant material and chemicals

Blue corn from the Mixteco maize variety was collected in the Mixteca region of Oaxaca, Mexico during 2012. For the chemical and biological analysis, sodium acetate, anhydrous sodium carbonate, potassium chloride, dimethyl sulfoxide (DMSO), folin reagent, gallic acid, ethanol, potassium acetate, quercetin, iron(III) chloride, hydrochloric acid, trolox, 2,2-difenil-1-picril-hidrazil (DPPH), amberlite XAD-7, acetic acid, 3-(4,5-dimethylthiazol-2-yl)-2,5-diphenyltetrazolium bromide (MTT) were obtained from Sigma-Aldrich. Trypsin, fetal bovine serum (FBS), GlutaMAX (100×) were purchased from Gibco. Dulbecco’s Modified Eagle’s medium (DMEM), Roswell Park Memorial Institute Medium (RPMI-1640) and phosphate buffer saline (PBS) tablet were supplied by Lonza.

### Tortilla preparation

Traditional nixtamalization was done by boiling blue corn in a solution of 1% calcium hydroxide at 92°C for 35 min. After standing for 16 h, the cooked blue corn nixtamal was rinsed three times with 1 L of purified water. Then it was grounded in a manual grinder to obtain dough. A domestic press was used to make tortillas with a thickness of 1 ± 0.5 mm, 12 ± 0.5 cm in diameter and 17.5 ± 0.5 g in weight. Discs of dough were put into a pan at 240 ± 2 °C for 30 s on the side A, followed by 65 s on side B, and 30 s again on the side A.

### Blue corn and tortilla extracts

Ground blue corn or tortilla (1:5 p:v) were homogenized with ethanol acidified with citric acid 1M. This was performed using an ultrasonic homogenizer (20 kHz, 750 W, Cole-Palmer Instrument Company, VCX-750, USA). The sample was placed under refrigeration for 24 h and centrifuged at 4000 rpm for 15 min at a temperature of 5°C. The process was repeated twice and the extract was concentrated using a rotary evaporator under vacuum. Extensive extractions were performed in order to obtain the highest content of polyphenols and anthocyanins. The conditions of extraction have been included in a patent request, MX/A/20131011202.

### Total phenolic content

The Folin–Ciocalteau method modified by Singleton and Rossi [13], was used to evaluate the total polyphenol content. The absorbance was measured at 750 nm. The total phenolic content was expressed as milligram equivalents of gallic acid/100 g of fresh weight (mg GAE/100 g FW).

### Total anthocyanin content

Monomeric anthocyanins were quantified using the pH differential method previously described by Giusti and Wrolstad [14]. Samples were diluted with 0.025 M potassium chloride buffer solutions at pH 1 and 0.4 M sodium acetate buffer at pH 4.5. A 400–700 nm sweep was done by using a spectrophotometer (PerkinElmer model Lambda 25 UV/VIS, USA). The monomeric anthocyanins content was expressed as mg of cyanidin-3-glucoside (C3G) per 100 g sample based on a molar extinction coefficient of 26,900 L cm^−1^ mg^−1^ and a molecular weight of 449.2 g/L.

### Antioxidant activity by DPPH

Antioxidant activity was determined by the DPPH method [15]. A standard calibration curve was established using trolox as the standard (100–800 µM). The radical DPPH (2.9 mL) was added to 0.1 mL of each extract. The mixture was incubated for 30 min in total darkness and the absorbance was read at 517 nm. The results were expressed in µmol equivalents of trolox g^−1^ of the sample (ET).

### TBARS assay

The evaluation of lipid peroxidation was performed by the TBARS method following the methodology described by Ohkawa [16]. For this, 400 µL of homogenized rat brain were mixed with 50 µL of extracts (500–1000 µg/mL) and incubated for 30 min at 37 °C. Lipid peroxidation with 50 µL of FeSO4 100 µM was induced, and after 1 h at 37° C, 500 µL of TBA reagent was added and the absorbance at 540 nm was measured.

### Isolation and chromatography analysis

A 45 × 1.5 cm column was packed with amberlite XAD-7 pre-conditioned with acidified water (5% acetic acid) [17]. The resin was washed with 200 ml of acidified water (5% acetic acid) and 1 mL of the blue corn or tortilla crude extract was added placed and washed with 100 mL of acidified water; the polymer mixture was eluted with 200 mL of acidified ethanol (5% acetic acid). The eluate was concentrated in a rotary evaporator (Heidolph Digital Laborota pump 4011 coupled to V Pimo Vacum Buchi 700) at 28 °C and stored at 4 °C until use. Separation of the compounds was performed using an HPLC equipped with a C-18 ZORBAX eclipse plus column (100 mm × 2.1 mm, 3.5 μm) under isocratic elution with methanol: water (2:8 v:v). The HPLC system was coupled to a Brüker MicrOTOF II spectrometer operating in negative ion mode, scan range: 50–3000 amu, capillary voltage 3.8 kV, dry gas flow at 4.0 L min^−1^ and heated capillary temperature of 180 °C. Under these conditions an electrospray ionization-mass spectrometry (ESI-MS) analysis of the isolated compounds was performed.

### MTT assay

Hepatocellular carcinoma (HepG2), lung carcinoma (H-460), cervix adenocarcinoma (Hela), breast adenocarcinoma (MCF-7), and androgen-independent prostate adenocarcinoma (PC-3) human cancer cell lines were obtained from the American Type Culture Collection, United States of America (ATCC, U.S). PC-3 cells were maintained in RPMI-1640 medium which contained 10% FBS, 1% l-glutamine and 0.1% piruvate. HepG2, Hela, H460 and MCF-7 cells were maintained in DMEM medium which contained 10% FBS. The antiproliferative action of ethanolic extracts of tortilla and blue maize of the Mixteco variety was evaluated in HepG2, H460, Hela, MCF-7 and PC-3 cancer cell lines. For this, cells in 96-well plates were grown to 80% confluency. The cell count was performed using a Neubauer chamber, 5000 cells were seeded per well for PC-3 and 10,000 cells per well for HepG2, H-460, Hela and MCF-7. The blue corn and tortilla extracts were applied in concentrations of 125, 250, 500 and 1000 µg/mL, 100 µL per well. Cell culture in medium was used as a negative control, R (−), and quercetin 50 µM as a positive control, R (+). Extracts and quercetin were solubilized in culture medium. Treatments were allowed to incubate for 48 h. Post incubation time cells were treated with 5 mg/mL of MTT (10 µL per well) and incubated at 37 °C, 5% CO_2_ for 2 h. Finally, 100 µL of DMSO were added, the absorbance was determined at 595 nm [18].

### Statistical analysis

Data analysis was performed using the GraphPad Prism 6.0 statistics software. Analysis of variance (ANOVA) and Tukey’s test comparison with a 95% confidence interval were performed. The results of cell viability are presented as mean ± SD of independent experiments.

## Results and discussion

### Total content polyphenols, monomeric anthocyanins, percent polymeric color and antioxidant activity in blue corn and tortilla

In the present study, the content of total polyphenols, monomeric anthocyanins and percent polymeric color in the grain of blue corn and the tortilla derived from it were evaluated, since it is relevant to know how the tortilla preparation process affects the concentration of phenolic compounds such as anthocyanins. The total phenolics value for blue corn was of 287.3, and 70.3 mg GAE/100 g for tortilla; while the monomeric anthocyanins content was 70.50 and 27.8 mg C3G/100 g in blue corn and tortilla, respectively (Table 1). According to literature data, a decrease in the concentration of polyphenols and anthocyanins in tortilla could be attributed to the nixtamalization process, were high temperature and alkaline conditions are applied [11, 12]. It has been estimated that 40–80% of anthocyanins may be lost during the transformation of blue corn grain to tortilla [19]. However, research made on blue corn tortilla show that the remaining amount of anthocyanins is enough to maintain antioxidant properties [11].

**Table 1:** Total polyphenol content, monomeric anthocyanins and antioxidant activity by DPPH and TBARS methods of blue corn and tortilla

Sample^1^	Total polyphenols (mg EAG/100 g)	Monomeric anthocyanins (mg C3G/100 g)	Percent polymeric color	DPPH (μM ET/g)	TBARS IC50 value (μg/mL)
Blue corn	287.3 ± 0.03^a^	70.50 ± 1.3^a^	54.0 ± 2.06^a^	49.2 ± 0.18^a^	792 ± 64.4^a^
Blue tortilla	70.3 ± 0.03^b^	27.8 ± 1.8^b^	66.1 ± 0.31^b^	45.1 ± 0.22^a^	750 ± 5.61^a^

*GAE* gallic acid equivalentes, *ET* trolox equivalente, *C3G* cyanidin 3-glucoside

^1^The results are expressed as mean ± SD. Different letters indicate that there are significant differences (p ≤ 0.05)

In addition, higher percent polymeric color values were observed for tortilla as compared to blue corn, which suggests the formation of polymers during the process. A study done by Lao and Giusti [20] suggests that percent polymeric color can be used as indicator of the effect of corn processing in anthocyanins. On the other hand, previous studies in thermally processed foods suggest that an increase in anthocyanin polymers has a positive effect on antioxidant activity [21].

On this regard, we used the DPPH and TBARS assays to provide information on the antioxidant activity of blue corn and tortilla extracts. The radical-scavenging activity by DPPH of these extracts is shown in Table 1. Interestingly, although tortilla extract showed a decrease on phenolics, it had slightly lower values of DPPH (45.1 μM ET/g FW) than blue corn (49.2 μM ET/g FW). Similar data were obtained by the TBARS assay (Table 1). The concentration of blue corn extract required to inhibit TBARS production by 50% (IC50) was slightly lower (750 µg/mL) than the one recorded for tortilla extract (792 µg/mL). These results suggest that changes in phenolic compounds such as anthocyanins on blue corn due to the nixtamalization process may improve its biological properties.

### HPLC–ESI-MS

**Fig. 1:**
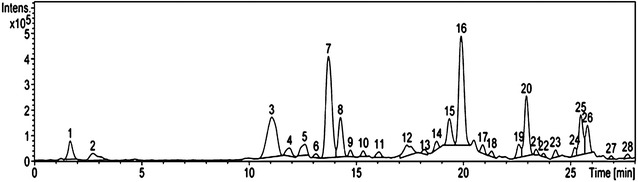
Anthocyanin profile of blue tortilla obtained by means of HPLC–ESI-MS

The anthocyanin composition of blue tortilla (Fig. 1) was estimated by HPLC coupled to mass spectrometry. A total of 28 compounds derived from cyanidin were tentatively identified in the blue tortilla extract (Table 2), including cyanidin-3-glucoside, four acylated anthocyanins and fifteen proanthocyanidins. It has been reported that during the process of nixtamalization, an alkaline hydrolysis takes place liberating the acid part of acylated anthocyanins, resulting in monoglycosylated forms of anthocyanins such as cyanidin-3-glucoside [19]. Previous studies point out that anthocyanins in blue maize grain are mainly acylated, which are more stable to alkaline pH and high temperatures [7, 22].

**Table 2:** Identification of anthocyanins in tortilla extract from blue Mixteco maize

Peak	RT (min)	[M]^+^ (m/z)	Fragments ions (m/z)	Compound
1	1.7	611	449, 287	Cyanidin-3,5-diglucoside
2	2.7	449	287	Cyanidin-3-glucoside
3	11.1	493	287	Unidentified
4	11.9	563	287	Cyanidin-3-(6-ethylmalonylglucoside)
5	12.6	565	287	Cyanidin-3-(malonyl)glucoside
6	13.1	735	460, 287	Unidentified
7	13.7	737	460, 292	Unidentified
8	14.3	490	287, 162	Cyanidin-3-O-(6″-acetyl-galactoside)
9	14.7	520	287, 162	Cyanidin-3-O-(6″-piruvoyl-glucoside)
10	15.3	725	287, 162	Unidentified
11	16.1	615	510, 287, 162	Unidentified
12	17.4	789	589, 303, 152	Proanthocyanidin dimer
13	18.2	877	717, 597, 347	Unidentified
14	18.7	921	641, 471, 403, 303, 162	Proanthocyanidin dimer
15	19.3	843	685, 433, 287, 162	Proanthocyanidin dimer
16	19.9	903	729, 463	Unidentified
17	20.9	817	757, 617, 463, 287, 162	Proanthocyanidin tetramer
18	21.3	861	801, 669, 477, 287, 162	Proanthocyanidin trimer
19	22.6	871	801, 671, 606, 538, 425, 287, 162	Proanthocyanidin trimer
20	22.9	901	636, 538, 287, 176	Proanthocyanidin trimer
21	23.4	901	801, 666, 463, 375, 287, 176	Proanthocyanidin trimer
22	23.7	901	801, 655, 463, 287, 162	Proanthocyanidin trimer
23	24.3	843	637, 502, 417, 337, 162	Proanthocyanidin trimer
24	25.2	871	637, 467, 287, 176	Proanthocyanidin trimer
25	25.5	901	729, 597, 463, 325, 176	Proanthocyanidin trimer
26	25.8	901	843, 627, 463, 287, 176	Proanthocyanidin trimer
27	26.9	901	729, 635, 439, 299, 177	Proanthocyanidin trimer
28	27.6	885	725, 339, 154	Proanthocyanidin trimer

The data obtained by percent polymeric color reveal that polymerized compounds are present in blue tortilla (Table 2). Studies on chemical composition on blue tortilla from different maize varieties mainly report monomeric and acylated anthocyanins [19], while the presence of proanthocyanidins had not been observed. In this study, the number of anthocyanins detected and discrepancies in their profile with previous reports could be attributed to the maize variety, as well as to the nixtamal process, where anthocyainins may be degraded or react among themselves, or even polymerize at the given alkaline and temperature conditions. The extraction method may also have an impact on anthocyanin profile. For better anthocyanin extraction, previous reports recommend to use weak organic acids and low concentrations of strong acids such as hydrochloric acid (< 1.0%) [23]. A study done by Lao and Giusti [20] report that high concentration hydrochloric acid could break the glycosidic bond of monomeric anthocyanins and hydrolyze polymeric pigments into smaller molecules. For this reason, we used ethanol acidified with citric acid since organic acids decrease the decomposition of anthocyanins during the concentration of extracts [24, 25]. Furthermore, it has been reported that acidic conditions allow a higher extraction of proanthocyanidins by preventing autoxidation and decreasing their polar interactions with the cell wall [26]. On this regard, our research team has studied different samples of blue tortilla made from other maize varieties, where proanthocyanidins have also been detected (unpublished data). As far as we know, this is a first report of proanthocyanidins on blue tortilla.

It is important to say that although the tortilla showed a decrease in the content of anthocyanins as compared to the grain (Table 1), the chemical changes due to the tortilla preparation process may be beneficial from the biological activity standpoint, as suggested by the antioxidant activity assays that were made in the present study. It could be attributed to the chemical composition of anthocyanin profile of tortilla where the presence of monomeric anthocyanins and proanthocyanidins were detected.

### Antiproliferative activity

**Fig. 2:**
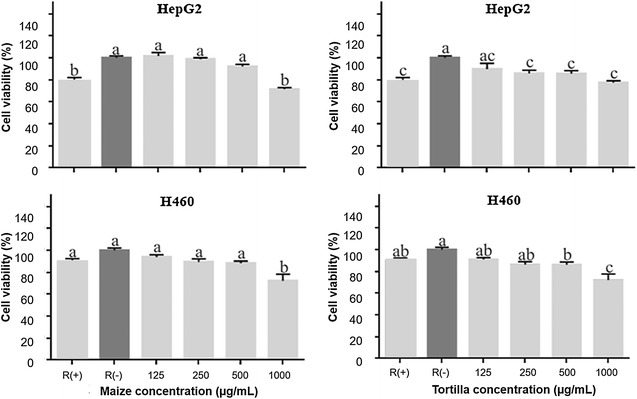
Effect of blue corn and tortilla extracts on cell viability in HepG2 and H460 human cancer cell lines. R (+). Quercetin 50 μ. R (−). Culture medium. Different letters indicate that there are significant differences (p ≤ 0.05)

**Fig. 3:**
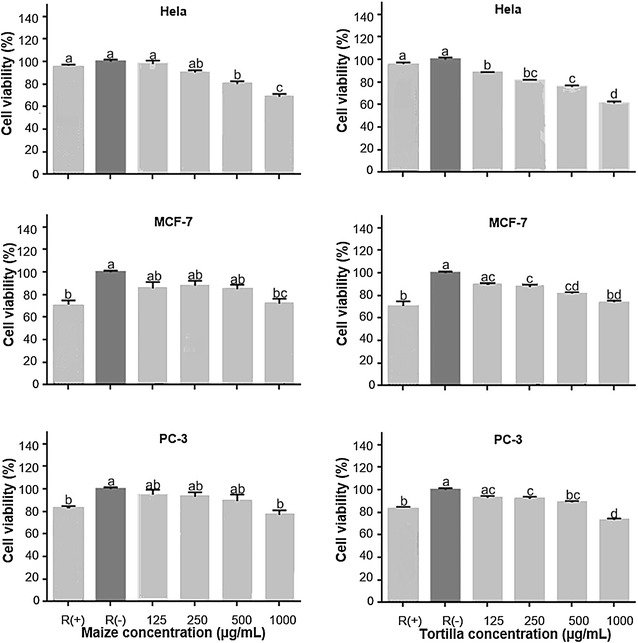
Effect of blue con and tortilla extracts on cell viability in Hela, MCF-7 and PC-3 human cancer cell lines. R (+). Quercetin 50 μM. R (−). Culture medium. Different letters indicate that there are significant differences (p ≤ 0.05)

In order to shed light on anticancer properties of blue corn and tortilla extracts, we investigated their antiproliferative activity on different cancer cell lines: hepatocellular carcinoma (HepG2), lung carcinoma (H-460), cervix adenocarcinoma (Hela), mammary adenocarcinoma (MCF-7) and prostate cancer androgen dependent (PC-3) as shown in Figs. 2, 3. These cell lines were selected because they represent the types of cancer with the highest incidence and mortality in Mexico [27].

For HepG2, H-460, MCF-7and PC-3 cancer cell lines, the blue corn extract significantly inhibited cell proliferation at 1000 µg/mL, (Figs. 2, 3). Interestingly, tortilla extract inhibited cell growth of these cancer lines at a lower concentration (250 and 500 μg/mL). However, blue corn as well as tortilla extracts exhibited the highest antiproliferative activity against HepG2, H-460, MCF-7 and PC-3 at 1000 µg/mL. It can be also observed that for these cell lines both extracts showed similar antiproliferative activity at this concentration, suggesting that the tortilla process is beneficial. These results are consistent with the findings by TBARS assay (Table 1), where the blue corn and tortilla extracts decreased lipid peroxidation (IC50) at similar concentrations. These data are clearly interesting, since they imply that processing of blue corn into tortilla increases health benefits. Previously, other studies reported anthocyanin content, antioxidant activity and the profiles of these compounds for blue corn and tortilla; however, specialized research on anticancer properties of blue corn and tortilla are scarce.

On the other hand, among all the cancer cell lines tested, blue corn and tortilla extract showed the highest antiproliferative activity in Hela. It is noteworthy that tortilla extract (61.04 ± 1.9%) showed slightly lower cell viability than blue corn extract (68.69 ± 2.6%) for the concentration of 1000 μg/mL. It could be attributed to changes on anthocyanin profile during nixtamalization, which probably favors the antiproliferative activity of tortilla extract in the cell line Hela. This evidence highlights once again the importance of the traditional nixtamalization process to make tortilla, particularly its effect on anthocyanins, compounds with anticancer properties.

A study done by Bagchi et al. [28] found that grape seed proanthocyanidins offered more protection against free radical-induced lipid peroxidation compared to vitamin C, E and β-carotene. Also, research into the effect of strawberry extract enriched with ellagitannins and proanthocyanidins indicate that it was most cytotoxic against tumourigenic clones and lymphocytes [29].

The proanthocyanidins are phenolic compounds that have shown chemoprotective properties against oxygen free radicals and oxidative stress; however, the mechanisms behind their anti-cancer activity have not been completely elucidated. Some authors suggest that the complexity of proanthocyanidins allows them to interfere in signaling systems [30].

Nevertheless, several molecules present in foods, such as quercetin, can inhibit the growth of cancer cells [31]. Therefore, we compared the antiproliferative activity of blue corn and tortilla extracts against quercetin 50 µM in all studied cell lines. Our results provide evidence that blue corn and tortilla extracts have similar antiproliferative activity in HepG2, MCF-7 and PC-3 or even better results in Hela and H-460 cell lines than the ones given by quercetin 50 µM. These results are promising and provide new information on anticancer properties of nixtamalization products, specifically tortilla prepared from blue corn.

## Conclusion

Our study is the first report showing that traditional tortilla-making process from blue corn favors antiproliferative activity in several cancer cell lines. This evidence highlights the importance of the traditional nixtamalization process, particularly its effect on anthocyanins. The findings suggest that consumption of blue corn and tortilla could have a positive effect on health. Further investigation is required to clarify the molecular mechanism(s) involved in the anticancer activity observed in blue corn and tortilla from maize of the Mixteco race.

## References

[1] Cassidy E, Mukamal KJ, Liu L, Franz M, Eliassen AE, Rimm E (2017). High anthocyanin intake is associated with a reduced risk of myocardial infarction in young and middle-aged women. Circulation.

[2] Delgado-Vargas F, Paredes-López O (2002) Anthocyanins and betalains. In: Natural colorants for food and nutraceutical uses. CRC, Boca Ratón, pp 167–191

[3] Abdel-Aal ESM, Awika JM, Piironen V, Bean S (2011). Anthocyanin-pigmented grain products. Advances in cereal science: implications to food processing and health promotion.

[4] Kato T, Mapes C, Mera L, Serratos J, Bye R (2009). Origen y diversificación del maíz: una revisión analítica.

[5] FAO (2016) Food and Agriculture Organization. http://faostat.fao.org

[6] López-Martinez LX, Parkin KL, Garcia HS (2014). Antioxidant and quinone reductase inducing activities of ethanolic fractions from purple maize. LWT-Food Sci Technol.

[7] Salinas-Moreno Y, Pérez-Alonso JJ, Vázquez-Carrillo G, Aragón-Cuevas F, Velázquez-Cardelas GA (2012). Antocianinas y actividad antioxidante en maíces (Zea mays L.) de las razas chalqueño, elotes cónicos y bolita. Agrociencia.

[8] Wang LS, Stoner GD (2008). Anthocyanins and their role in cancer prevention. Cancer Lett.

[9] Kong JM, Chiam SL, Goh NK, Chia TF, Brouillar C (2003). Analysis and biological activities of anthocyanins. Phytochemistry.

[10] FAO (1992) Maize in human nutrition. FAO food and nutrition series

[11] De la Parra C, Serna-Saldivar SO, Liu RH (2007). Effect of processing on the phytochemical profiles and antioxidant activity of corn for production of masa, tortillas, and tortilla chips. J Agric Food Chem.

[12] Cortes GA, Salinas MY, Martín-Martínez SE, Martínez-Bustos F (2006). Stability of anthocyanins of blue maize (Zea mays L.) after nixtamalization of separated pericarp-germ tip cap and endosperm fractions. J Cereal Sci.

[13] Singleton VL, Rossi JA (1965). Colorimetry of total phenolics with phosphomolybdic phosphotungstic acid reagents. Am J Enol Vitic.

[14] Giusti M, Wrolstad RE, Giusti MM, Wrolstad RE (2001). Characterization and measurement of anthocyanins by UV visible spectroscopy. Current protocols in food analytical chemistry.

[15] Brand-Williams W, Cuvelier ME, Berset C (1995). Use of a free radical method to evaluate antioxidant activity. LWT-Food Sci Technol.

[16] Ohkawa H, Ohishi N, Yagi K (1979). Assay for lipid peroxides in animal tissues by thiobarbituric acid reaction. Anal Biochem.

[17] Welch CR, Wu Q, Simon JE (2008). Recent advances in anthocyanins analysis and characterization. Curr Anal Chem.

[18] Mosmann T (1983). Rapid colorimetric assay for cellular growth and survival: application to proliferation and cytotoxicity assays. J Immunol Methods.

[19] Salinas-Moreno Y, Martinez-Bustos F, Soto-Hernandez M, Ortega-Paczka R, Arellano-Vazquez JL (2003). Effect of alkaline cooking process on anthocyanins in pigmented maize grain. Agrociencia.

[20] Lao F, Giusti MM (2016). Quantification of purple corn (Zea mays L.) anthocyanins using spectrophotometric and HPLC approaches: method comparison and correlation. Food Anal Methods.

[21] Brownmiller C, Howard LR, Prior RL (2008). Processing and storage effects on monomeric anthocyanins, percent polymeric color, and antioxidant capacity of processed blueberry products. J Food Sci.

[22] Guzmán-Gerónimo RI, Alarcón-Aparicio E, García-Barrradas O, Alarcón-Zavaleta T, Chávez-Servia JL, Alarcón-Zavaleta Tania, Badria FA (2017). Cytotoxic activity of blue corn extract on several cancer cell lines. Phytochemistry natural products and cancer.

[23] Nicoué EE, Savard S, Belkacemi K (2007). Anthocyanins in wild blueberries of quebec: extraction and identification. J Agric Food Chem.

[24] Hosseini S, Gharachorloo M, Ghiassi-Tarzi B, Ghavami M (2016). Evaluation of the organic acids ability for extraction of anthocyanins and phenolic compounds from different sources and their degradation kinetics during cold storage. Pol J Food Nutr Sci.

[25] Dao LT, Takeoka GR, Edwards RH, Berrios JDJ (1998). Improved method for the stabilization of anthocyanidins. J Agric Food Chem.

[26] Gu L, Xu Z, Howard LR (2012). Analysis methods of proanthocyanidins. Analysis of antioxidant-rich phytochemicals.

[27] SS (2016) Secretaría de salud de México. http://www.dgis.salud.gob.mx/contenidos/sinais/e_mortalidadgeneral.htm

[28] Bagchi D, Garg A, Krohn RL, Bagchi M, Tran MX, Stohs SJ (1997). Oxygen free radical scavenging abilities of vitamins C and E, and a grape seed proanthocyanidin extract in vitro. Res Commun Mol Pathol Pharmacol.

[29] Weaver J, Briscoe T, Hou M, Goodman C, Kata S, Ross H, McDougall G, Stewart D, Riches A (2009). Strawberry polyphenols are equally cytotoxic to tumourigenic and normal human breast and prostate cell lines. Int J Oncol.

[30] Nandakumar V, Singh T, Katiyar SK (2009). Multi-targeted prevention and therapy of cancer by proanthocyanidins. Cancer Lett.

[31] Duo J, Ying GG, Wang GW, Zhang L (2012). Quercetin inhibits human breast cancer cell proliferation and induces apoptosis via Bcl-2 and Bax regulation. Mol Med Rep.

